# High frequency of Voltage-gated sodium channel (*VGSC*) gene mutations in *Aedes albopictus* (Diptera: Culicidae) suggest rapid insecticide resistance evolution in Shanghai, China

**DOI:** 10.1371/journal.pntd.0011399

**Published:** 2023-06-02

**Authors:** Hao Yuan, Wenqi Shan, Yuhang Zhang, Hanlu Yan, Yikai Li, Qiuming Zhou, Haowei Dong, Feng Tao, Hongxia Liu, Peien Leng, Heng Peng, Yajun Ma

**Affiliations:** 1 Department of Naval Medicine, Naval Medical University, Shanghai, China; 2 The 1^st^ Cadet Corp, College of Basic Medical Science, Naval Medical University, Shanghai, China; 3 Department of Pathogen Biology, College of Basic Medical Sciences, Naval Medical University, Shanghai, China; 4 Shanghai Municipal Center for Disease Control and Prevention, Shanghai, China; Universita degli Studi di Pavia, ITALY

## Abstract

**Background:**

Dengue fever is an infectious disease that is imported into Shanghai, China and requires prevention and control measures. Controlling the vector *Aedes albopictus* through insecticide use is a key approach to dengue control. However, the rapid evolution of insecticide resistance in *Ae*. *albopictus* has raised concerns about the failure of dengue control efforts. Knockdown resistance (*kdr*) caused by point mutations in the voltage-gated sodium channel (*VGSC*) gene is a primary mechanism of pyrethroid resistance. In this study, we investigated the *kdr* mutations of *Ae*. *albopictus* in Shanghai and evaluated the trend in its evolution.

**Methodology/principal findings:**

We collected 17 populations of *Ae*. *albopictus* from 15 districts in Shanghai in 2020, extracted genomic DNA from individual mosquitoes, and amplified Domain II, III, and IV in *VGSC* using PCR. Following sequencing, we obtained 658 *VGSC* sequences. We detected the nonsynonymous mutations V1016G, I1532T, and F1534S/C/I, among which V1016G and F1534C/I were reported in Shanghai for the first time and F1534I was a novel mutant allele in *Ae*. *albopictus*. The overall mutation frequency was 84.65%, with individual mutation frequencies ranging from 46.81% to 100%, excluding the Fengxian District population, which had a frequency of 0%. The V1016G and I1532T mutation types accounted for 7.14% and 3.42%, respectively. The mutant allele at codon 1534 accounted for 63.98% of all mutations, including TCC/S (62.77%), TGC/C (1.06%), and ATC/I (0.15%). We identified and classified five intron types in Domain III by length, including A (83 bp, 12.07%), B (68 bp, 87.30%), C (80 bp, 0.16%), D (72 bp, 0.16%), and E (70 bp, 0.31%). Individuals with intron B had a significant mutation tendency at codon 1534 relative to intron A (chi-square test, *p* < 0.0001). We found no correlation between mutation frequency and the amount of pyrethroid used (Pearson correlation, *p* = 0.4755).

**Conclusions/significance:**

In recent years, *kdr* mutations in the *Ae*. *albopictus* population in Shanghai have rapidly evolved, as evidenced by an increase in mutation types and significantly increased mutation frequency. The F1534I/ATC mutant allele was found to be a novel mutation, F1534C/TGC was reported for the first time in Shanghai, and intron B in Domain III was significantly associated with mutation frequency at codon 1534. Continuous monitoring of resistance changes and strict regulation of insecticide use are required.

## Author summary

*Aedes albopictus* is the primary dengue fever vector in Shanghai, China. Given the lack of an effective vaccine, vector control remains the key prevention method for this infectious disease. Pyrethroids are the preferred insecticide for vector control owing to their high efficiency and low toxicity; however, vector resistance has reached a high level under selection pressure. Pyrethroids target the voltage-gated sodium channel (VGSC), and mutations in the coding region of the *VGSC* gene can result in knockdown resistance (*kdr*), one of the primary mosquito resistance mechanisms. This study explored *kdr* mutations in 17 *Ae*. *albopictus* populations collected from 15 districts in Shanghai in 2020. The overall mutation frequency was alarmingly high, reaching 84.65%. Mutant *VGSC* alleles, including V1016G, I1532T, and F1534S/C/I, were detected, with V1016G and F1534C/I being reported for the first time in Shanghai and F1534I being a novel mutant allele in *Ae*. *albopictus*. Additionally, five types of introns in Domain III were identified, with intron B type found to be significantly associated with mutation frequency at codon 1534. The rapid evolution trend of *kdr* mutations is concerning and indicates a growing risk of mosquito-borne disease outbreaks. Therefore, continuous monitoring of resistance changes and strict regulation of insecticide use are necessary.

## Introduction

Shanghai, with a population of over 20 million people and a high number of annual migrants, faces a high risk of imported infectious diseases [1–5], including dengue fever [6], a mosquito-borne disease that is prevalent worldwide [7]. In 2017, a local case of dengue fever was reported in Shanghai, which was caused by secondary transmission from imported cases [8]. *Aedes albopictus* is the main vector of dengue fever in mainland China, having been found in 25 provinces (municipalities) [9]. In particular, *Ae*. *albopictus* is the main vector of dengue fever in Shanghai, as *Aedes aegypti* is not distributed in the area. With no effective vaccine for dengue fever, controlling the mosquito vector population is the primary prevention method [10–12]. Chemical insecticides are a preferred mosquito control method, particularly pyrethroids owing to their efficiency and low toxicity [13]. However, overuse of pyrethroids leads to selection pressure toward mosquitoes, resulting in significant resistance development and global public health concerns arising from uncontainable outbreaks of mosquito-borne diseases [14]. Therefore, it is crucial to monitor mosquito resistance, particularly in areas with a high risk of dengue fever outbreaks.

Mosquitoes have developed four insecticide resistance mechanisms that have been categorized as metabolic resistance, target-site resistance, behavioral resistance, and reduced penetration. Metabolic and target-site resistance are considered the most significant factors contributing to resistance [14]. Target-site resistance arises from genetic changes in the coding region of a locus that modifies protein structure and function, leading to a decrease or prevention of insecticide binding at action sites. Knockdown resistance (*kdr*) is a crucial target-site resistance mechanism commonly observed in pyrethroid resistance in insects [15]. *Kdr* is the result of mutations in the voltage-gated sodium channel (VGSC) encoded by the *VGSC* gene, which is the pyrethroid action site [16].

In 2009, Kasai *et al*. first reported the F1534C mutation in *Ae*. *albopictus* collected in Singapore, marking the first discovery of *kdr* mutations in this population [17]. Since then, various mutations, including V1016G/I, I1532T, and F1534S/C/L, have been detected in different parts of the world [18–21]. In China, our research group first reported the presence of F1534S and F1534L in Haikou, Hainan Province, with F1534S thought to be associated with pyrethroid resistance [22]. Subsequently, F1534S, F1534C, F1534L, and I1532T were detected in Hainan, Guangdong, Jiangsu, Shandong and other provinces [23–25]. V1016G was found in Yunnan, Beijing, and Guangdong [26–28], whereas D1763Y was only found in Ruili, Yunnan Province [29]. In Shanghai, a significant amount of insecticide has been used to control mosquitoes, which has led to a certain degree of resistance. Gao *et al*. [30] found that two populations of *Ae*. *albopictus* collected from Baoshan and Yangpu districts in Shanghai in 2017 were both resistant to pyrethroids and had the *kdr* mutations I1532T and F1534S. However, information regarding *kdr* mutations in *Ae*. *albopictus* populations in Shanghai is scarce in the literature.

To assess the prevalence of *kdr* mutation in *Ae*. *albopictus* populations in Shanghai, we collected 17 populations from 15 districts in 2020 and amplified 3 *VGSC* gene fragments to detect *kdr* mutations. The high frequency of *kdr* mutations and the identification of new mutant alleles suggest the rapid evolution of insecticide resistance in *Ae*. *albopictus* in Shanghai. These findings highlight an increased risk of dengue outbreaks and emphasize the need for stricter regulations on the use of insecticides.

## Materials and methods

### Sample collection and identification

**Fig 1:**
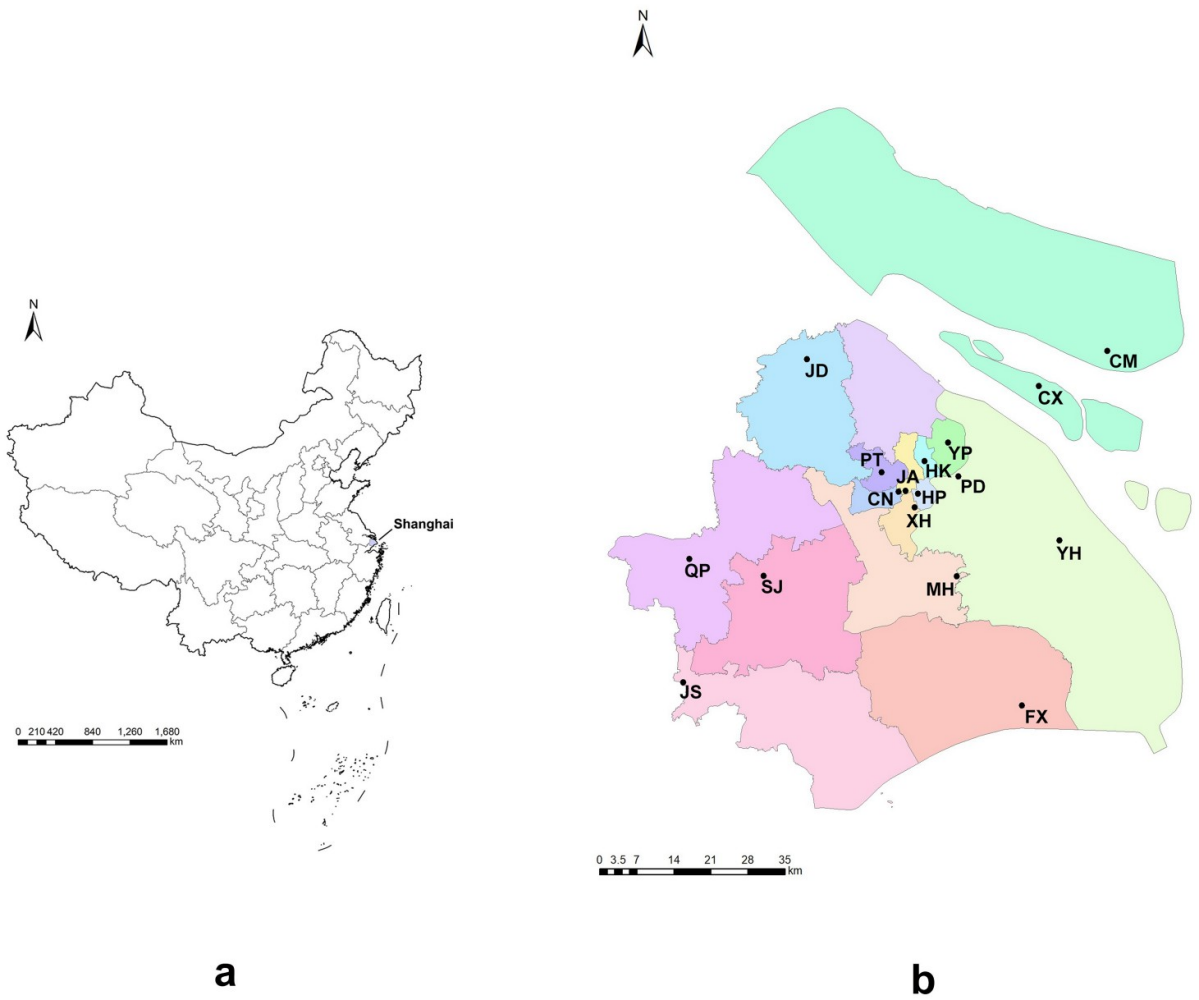
*Aedes albopictus* sample collection sites in Shanghai, China. a. Schematic map of China showing the location of Shanghai. b. Schematic map of Shanghai showing sampling sites. CM, Chongming District; FX, Fengxian District; QP, Qingpu District; XH, Xuhui District; SJ, Songjiang District; PT, Putuo District; YH, Yuanhang Road in Pudong District; JS, Jinshan District; JD, Jiading District; HK, Hongkou District; MH, Minhang District; CX, Changxing Island in Chongming District; PD, Pudong District; JA, Jing’an District; YP, Yangpu District; CN, Changning District; HP, Huangpu District. The map was made using ArcGIS 10.8 (https://www.arcgis.com/) and Adobe Photoshop 2020. The basemap source was the National Platform for Common Geospatial Information Services (https://www.tianditu.gov.cn/)).

Mosquito populations were collected from 17 sites in 15 districts of Shanghai between May to October 2020 (Fig 1; Table 1). Larvae and pupae found in stagnant water contained in tires, waste containers, receptacles, and jardinieres were collected using straws and brought back to the laboratory, where they were reared to adults under standard conditions (26°C ± 1°C, 65% ± 5% relative humidity, 12/12 h day/night photoperiod). Each sampling site contained more than 10 breeding sites, and a few adult samples were captured using aspirators. The emerging mosquitoes were collected within 24 h and then killed by freezing. After morphological identification to confirm the *Ae*. *albopictus* species [31], the mosquitoes were either stored at −80°C or subjected to genome extraction immediately.

**Table 1:** The collection information of *Ae*. *albopictus* in Shanghai, China.

Districts	Code	Collection site	Longitude/latitude	Environment	Date
Pudong	PD	Linggao Community	121.550E/31.247N	waste container, receptacles and jardinieres	2020/10/2
Fengxian	FX	Shanghai Bay National Forest Park	121.691E/30.862N	tyres, waste containers	2020/10/8
Putuo	PT	Shanghai Sports Palace	121.397E/31.252N	tyres, waste containers, receptacles and jardinieres	2020/10/14
Jinshan	JS	Fengjing Town	121.017E/30.887N	tyres, waste containers	2020/10/8
Jing’an	JA	Jing’an Park	121.447E/31.222N	stagnant water, waste containers	2020/10/10
Songjiang	SJ	Xiayang Village	121.166E/31.073N	waste containers	2020/10/9
Changxing Island (Chongming)	CX	Changxing Island Country Park	121.710E/31.403N	tyres, waste containers	2020/9/29
Minhang	MH	The Grain Calling Mansion	121.550E/31.073N	waste containers	2020/10/13
Hongkou	HK	Luxun Park	121.483E/31.272N	waste containers, receptacles and jardinieres	2020/9/23
Qingpu	QP	Shanghai Oriental Land	121.011E/31.105N	tyres, waste containers	2020/10/9
Chongming	CM	Yingdong Village	121.843E/31.464N	waste containers, receptacles and jardinieres	2020/10/11
Jiading	JD	Taqiao Rose Cultural Park	121.246E/31.442N	waste containers	2020/10/12
Yuanghang Road (Pudong)	YH	Sifan South Garden	121.754E/31.140N	waste containers	2020/10/18
Huangpu	HP	Fuxing Park	121.469E/31.217N	waste container	2020/10/10
Xuhui	XH	Jiangnan New Village	121.463E/31.192N	waste container	2020/10/16
Changning	CN	Jiangsu Street	121.431E/31.218N	waste container	2020/9/22
Yangpu	YP	Naval Medical University	121.524E/31.306N	tyres	2020/5/26

### Genomic DNA extraction

Genomic DNA was extracted from a single adult mosquito using a spin-column DNA extraction technique via DNA extraction kits (Aidlab, Beijing, China) following the manufacturer’s instructions. The extracted DNA was stored at −20°C for future use.

### PCR amplification and sequencing of *VGSC* fragments

The *VGSC* gene of *Ae*. *albopictus* contains four domains (I–IV), and nonsynonymous point mutations have been found only in Domain II, III, and IV [17, 32]. To detect point mutations in *VGSC*, fragments from Domain II, III, and IV were amplified via PCR under the same conditions. The 20 μL reaction mixture contained genomic DNA as templates, 10 μL of premix *Taq* (Aidlab, Beijing, China), 0.5 μL each of forward and reverse primers, 1 μL of genomic DNA, and 8 μL of ddH_2_O. The PCR reaction was conducted in a ProFlex PCR System (Applied Biosystems, Thermo Fisher Scientific), with the following cycling conditions: 94°C for 2 min, followed by 35 cycles at 94°C for 30 s, 60°C for 30 s, and 68°C for 30 s, with a final extension at 68°C for 8 min. The amplified products were detected using 1.5% agarose gel electrophoresis and sequenced using the Sanger sequencing method by BioSune Biotechnology Co., Ltd. The amplification and sequencing primers were the same and are listed in Table 2 [17].

**Table 2:** List of primers for PCR amplification of DNA sequences of Domain II, III, IV in *VGSC* gene [17].

Fragment	Primer		Sequence (5’- 3’)
Domain II	aegSCF3	Forward	GTGGAACTTCACCGACTTCA
	aegSCR22	Reverse	TTCACGAACTTGAGCGCGTTG
Domain III	aegSCF7	Forward	GAGAACTCGCCGATGAACTT
	albSCR9	Reverse	CTGATCCTCCGTCATGAACA
Domain IV	albSCF6	Forward	TCGAGAAGTACTTCGTGTCG
	albSCR8	Reverse	AACAGCAGGATCATGCTCTG

To distinguish the aberrant peaks in the sequence data, TA cloning was performed on the PCR products of several individuals for Domain III using a pMD 18-T Vector Kit (TaKaRa, Dalian, China) following the manufacturer’s instructions for cloning and sequencing.

### Detection and analysis of *kdr* mutations

The sequences were aligned and analyzed using DNASTAR Lasergene 12.0 software with partial sequences of an *Ae*. *albopictus* susceptible colony, including MT740753 of Domain II, MN433863.1 of Domain III, and MZ964134.1 of Domain IV, as references [33]. Excel 2019 (Microsoft, Redmond, WA, USA) was used to scan *kdr* mutation alleles and genotypes. SPSS version 21.0 (SPSS IBM Corp., Armonk, NY, USA) was used to analyze the allele and genotype frequencies of *VGSC* [34]. Any allele carrying nonsynonymous mutations (*kdr* mutation allele) was considered a mutant individual. The mutation frequency was calculated as the number of mutant individuals (wildtype/mutant heterozygotes + mutant genotypes) divided by the sample size.

### Detection of introns and correlation with *kdr* mutation genotypes

The length of introns in the *VGSC* gene was determined using DNASTAR Lasergene 12.0 based on the reference sequences mentioned above. The types of introns were classified by length and recorded in Excel 2019. It was not possible to accurately identify the introns in Domain II owing to the inconsistent sequencing lengths of forward and reverse strands. In Domain IV, no intron was found [17].

To analyze the correlation between introns and *kdr* genotypes in Domain III, Pearson correlation was performed using SPSS version 21.0. In addition, intron sequences from Domain III were obtained using TA cloning products and aligned using the MUSCLE algorithm in MEGA 11 (Mega Limited, Auckland, New Zealand) [35]. Sequence comparison and phylogenetic tree construction were also performed using MEGA 11.

### Application of pyrethroids in Shanghai and its correlation with *kdr* mutation frequency

The Shanghai Municipal Center for Disease Control and Prevention provided information on the types and quantities of pyrethroid insecticides used in each district of Shanghai in 2021. The total area of each district was obtained from Shanghai Municipal Statistics Bureau’s website (https://tjj.sh.gov.cn/tjnj/20220309/0e01088a76754b448de6d608c42dad0f.html). To investigate the correlation between pyrethroid insecticide usage and *kdr* mutation frequencies, the amount of insecticides used per unit area was calculated by dividing the total amount of pyrethroids by the area of each district. Pearson correlation analysis was performed using SPSS version 21.0.

### *Kdr* mutation history data of *Ae*. *albopictus* in Shanghai

To investigate the historical *kdr* mutation frequency in *Ae*. *albopictus* in Shanghai, data were obtained from Pubmed (https://pubmed.ncbi.nlm.nih.gov/) and CNKI (https://www.cnki.net/). The search term “*Ae*. *albopictus* and *kdr* and Shanghai” was used, covering the period from 2000 to 2022.

## Results

### Frequency of *kdr* mutations

After excluding the HP population due to a small sample size, *Ae*. *albopictus* was investigated in 16 collection sites (i.e., 16 populations) across 14 districts. In total, 658 individuals were analyzed for nonsynonymous mutations in Domain II, III, and IV fragments of *VGSC*. Nonsynonymous mutations were detected at codon 1016 (V1016G) of Domain II as well as codons 1532 (I1532T) and 1534 (F1534S/C/I) of Domain III, whereas no such mutations were found in Domain IV. In addition, *kdr* mutations were found in all populations, except for the FX population, with mutation frequencies ranging from 46.81% (QP) to 100% (XH/PT/MH/JS/JA) (Table 3). The mutation frequencies in JD, SJ, YH, CX, and CN were >90% whereas those in PD, YP, and HK were >80% (Table 3).

**Table 3:** The mutation frequency of *Ae*. *albopictus* in collection sites.

Code	Number of samples	Mutation frequency
FX	37	0.00%
QP	47	46.81%
CM	38	68.42%
PD	45	80.00%
YP	46	86.96%
HK	42	88.10%
JD	40	95.00%
SJ	42	95.24%
YH	37	97.30%
CX	38	97.37%
CN	40	97.50%
XH	40	100.00%
PT	37	100.00%
MH	40	100.00%
JS	39	100.00%
JA	50	100.00%
Total	658	84.65%

The mutation frequency was calculated as mutation individuals (wildtype/mutant heterozygotes + mutant genotypes) divided by the total number of samples.

### Frequency and distribution of *kdr* alleles at codon 1016

**Fig 2:**
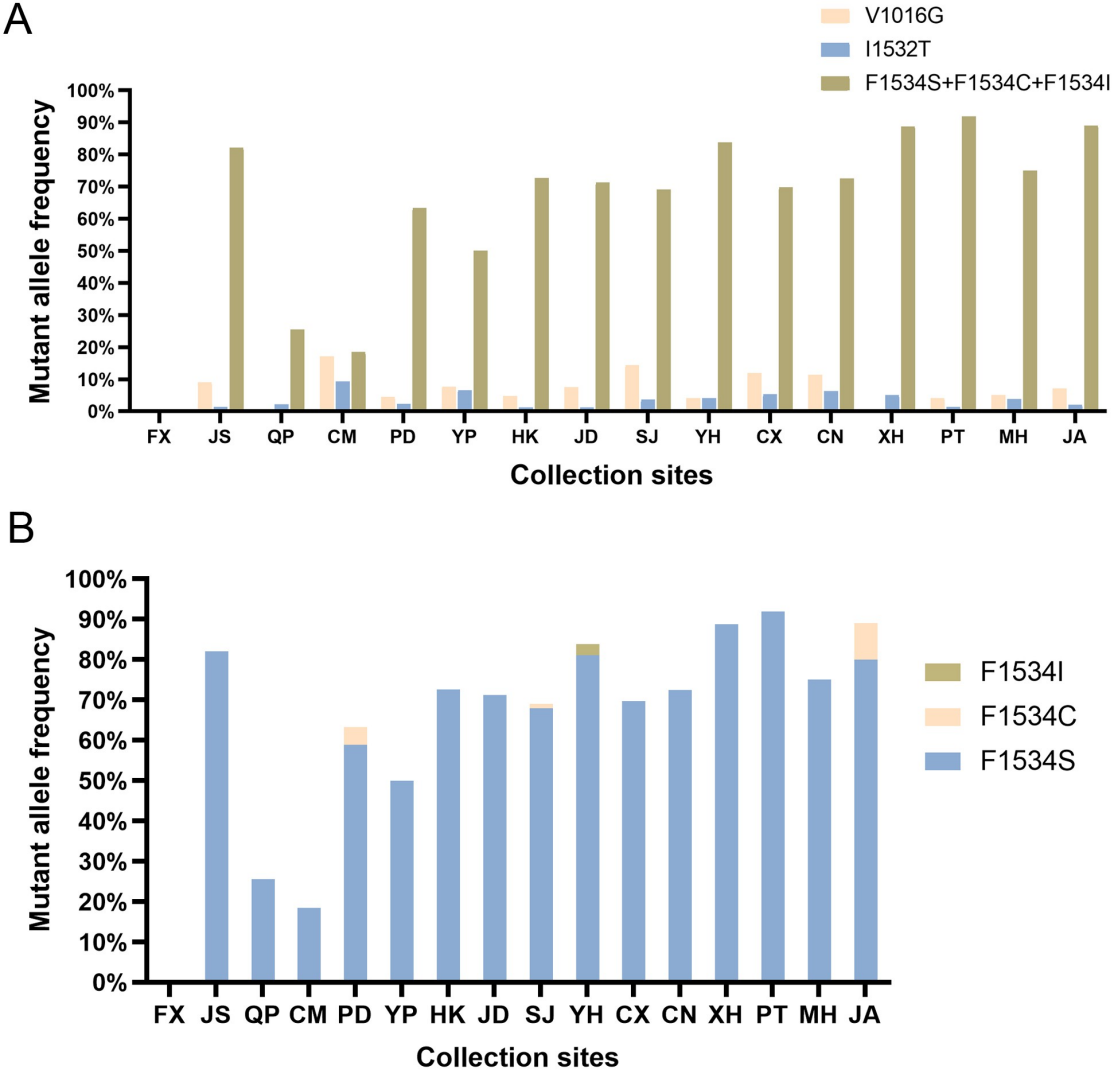
Histogram of mutant allele frequency in the *VGSC* gene of *Ae*. *albopictus* populations in Shanghai, China. A. Mutant allele frequencies at codon 1016, 1532, and 1534 of *VGSC*. Allele frequencies contained V1016G, I1532T, and the summed frequencies of F1534S, F1534C, and F1534I. B. Mutant allele frequency at codon 1534 of *VGSC*. FX, Fengxian District; JS, Jinshan District; QP, Qingpu District; CM, Chongming District; PD, Pudong District; YP, Yangpu District; HK, Hongkou District; JD, Jiading District; SJ, Songjiang District; YH, Yuanhang Road in Pudong District; CX, Changxing Island in Chongming District; CN, Changning District; XH, Xuhui District; PT, Putuo District; MH, Minhang District; JA, Jing’an District.

The wild-type allele at codon 1016 was GTA/V, and only the mutant GGA/G was detected. Herein, we report the presence of the V1016G allele in Shanghai for the first time. Three genotypes were identified: wild-type homozygous GTA/GTA (V/V), wild/mutant heterozygous GTA/GGA (V/G), and mutant homozygous GGA/GGA (G/G). V1016G was found in 13 populations, excluding FX, QP, and XH, in Shanghai. The mean frequency of the V1016G allele was 7.14%, ranging from 4.05% (YH) to 17.11% (CM). The wild-type genotype V/V was dominant, representing 86.63%, followed by wild/mutant heterozygous V/G genotype (12.46%) and mutant homozygous G/G genotype (0.91%) (Fig 2A; Table 4).

**Table 4:** Alleles and genotypes at codon 1016 of *VGSC* gene in *Ae*. *albopictus* from Shanghai China.

Code	N	Allele (%)		Genotype (%)		
		V/GTA	G/GGA	V/V	V/G	G/G
FX	37	100.00	0.00	100.00	0.00	0.00
QP	47	100.00	0.00	100.00	0.00	0.00
CM	38	82.89	17.11	71.25	23.68	5.26
PD	45	95.56	4.44	91.11	8.89	0.00
YP	46	92.39	7.61	84.78	15.22	0.00
HK	42	95.24	4.76	92.86	4.76	2.38
JD	40	92.50	7.50	85.00	15.00	0.00
SJ	42	85.71	14.29	73.81	23.81	2.38
YH	37	95.95	4.05	91.89	8.11	0.00
CX	38	88.16	11.84	76.32	23.68	0.00
CN	40	88.75	11.25	77.50	22.50	0.00
XH	40	100.00	0.00	100.00	0.00	0.00
PT	37	95.95	4.05	91.89	8.11	0.00
MH	40	95.00	5.00	90.00	10.00	0.00
JS	39	91.03	8.97	84.62	12.82	2.56
JA	50	93.00	7.00	88.00	10.00	2.00
Total/mean	658	92.86	7.14	86.63	12.46	0.91

N, number of samples.

### Frequency and distribution of *kdr* alleles at codon 1532

At codon 1532, only one mutant allele (ACC/T) was detected in addition to the wild-type allele (ATC/I). Three genotypes were identified: wild-type homozygous ATC/ATC (I/I), wild/mutant heterozygous ATC/ACC (I/T), and mutant homozygous ACC/ACC (T/T).

The mean mutation frequency of the mutant allele I1532T was 3.42%, with a range of 1.11% (JD) to 9.21% (CM), across all collection sites except FX. The wild-type homozygous genotype I/I was the dominant genotype, representing 93.62%, followed by the I/T (5.93%) and T/T (0.46%) genotypes (Fig 2A; Table 5).

**Table 5:** Alleles and genotypes at codon 1532 of *VGSC* gene in *Ae*. *albopictus* from Shanghai China.

Code	N	Allele (%)		Genotype (%)		
		I/ATC	T/ACC	I/I	I/T	T/T
FX	37	100.00	0.00	100.00	0.00	0.00
QP	47	97.87	2.13	95.74	4.26	0.00
CM	38	90.79	9.21	86.84	7.89	5.26
PD	45	97.78	2.22	95.56	4.44	0.00
YP	46	93.48	6.52	86.96	13.04	0.00
HK	42	98.81	1.19	97.62	2.38	0.00
JD	40	98.89	1.11	97.78	2.22	0.00
SJ	42	96.43	3.57	92.86	7.14	0.00
YH	37	95.95	4.05	91.89	8.11	0.00
CX	38	94.74	5.26	92.11	5.26	2.63
CN	40	93.75	6.25	87.50	12.50	0.00
XH	40	95.00	5.00	90.00	10.00	0.00
PT	37	98.65	1.35	97.30	2.70	0.00
MH	40	96.25	3.75	92.50	7.50	0.00
JS	39	98.72	1.28	97.44	2.56	0.00
JA	50	98.00	2.00	96.00	4.00	0.00
Total/mean	658	96.58	3.42	93.62	5.93	0.46

N, number of samples.

### Frequency and distribution of *kdr* alleles at codon 1534

At codon 1534, the wild-type allele was TTC/F. One novel synonymous mutation TTT/F was found, and three nonsynonymous mutations were detected: TCC/S, TGC/C, and ATC/I. Six genotypes were identified: wild-type homozygous TTC/TTC (F/F) and wild-type heterozygous TTC/TTT (F/F), wild-type/mutant heterozygous TTC/TCC (F/S) and TTT/TCC (F/S), mutant homozygous TCC/TCC (S/S) and TGC/TGC(C/C), and mutant heterozygous TCC/TGC (S/C) and TCC/ATC (S/I).

The wild-type allele at codon 1534 accounted for 36.02%, including allele TTC (35.87%) and TTT (0.15%). The frequency of the mutant allele F1534S was the highest (62.77%), whereas the other two mutant alleles, F1534C and F1534I, had low frequencies (1.06% and 0.15%, respectively) (Fig 2B; Table 6). The F1534S mutation was found at most collection sites except FX, and its frequency in 13 collection sites was ≥ 50%, ranging from 50.00% (YP) to 91.89% (PT), with frequencies of 25.53% and 18.42% in QP and CM, respectively. Herein, we report the mutant allele F1534C for the first time in Shanghai, where it was identified in three populations, namely SJ (1.19%), PD (4.44%), and JA (9.00%). In contrast, the F1534I mutation was found only in YH (2.70%).

**Table 6:** Alleles and genotypes at codon 1534 of *VGSC* gene in *Ae*. *albopictus* from Shanghai China.

Code	N	Allele (%)				Genotype (%)						
		F/TTC	F/TTT	S/TCC	C/TGC	I/ATC	F/F*	F/S*	S/C	S/I	S/S	C/C
FX	37	100.00	0.00	0.00	0.00	0.00	100.00	0.00	0.00	0.00	0.00	0.00
QP	47	74.47	0.00	25.53	0.00	0.00	55.32	38.30	0.00	0.00	6.38	0.00
CM	38	81.58	0.00	18.42	0.00	0.00	65.79	31.58	0.00	0.00	2.63	0.00
PD	45	36.67	0.00	58.89	4.44	0.00	26.67	20.00	0.00	0.00	48.89	4.44
YP	46	50.00	0.00	50.00	0.00	0.00	26.09	47.83	0.00	0.00	26.09	0.00
HK	42	27.38	0.00	72.62	0.00	0.00	16.67	21.43	0.00	0.00	61.90	0.00
JD	40	28.75	0.00	71.25	0.00	0.00	4.44	47.50	0.00	0.00	47.50	0.00
SJ	42	28.57	2.38	67.86	1.19	0.00	16.67*	28.57*	2.38	0.00	52.38	0.00
YH	37	16.22	0.00	81.08	0.00	2.70	2.70	27.03	0.00	5.41	64.86	0.00
CX	38	30.26	0.00	69.74	0.00	0.00	18.42	23.68	0.00	0.00	57.89	0.00
CN	40	27.50	0.00	72.50	0.00	0.00	10.00	35.00	0.00	0.00	55.00	0.00
XH	40	11.25	0.00	88.75	0.00	0.00	2.50	17.50	0.00	0.00	80.00	0.00
PT	37	8.11	0.00	91.89	0.00	0.00	0.00	16.22	0.00	0.00	83.78	0.00
MH	40	25.00	0.00	75.00	0.00	0.00	2.50	45.00	0.00	0.00	52.50	0.00
JS	39	17.95	0.00	82.05	0.00	0.00	5.13	25.64	0.00	0.00	69.23	0.00
JA	50	11.00	0.00	80.00	9.00	0.00	2.00	18.00	18.00	0.00	62.00	0.00
Total/mean	658	35.87	0.15	62.77	1.06	0.15	22.04	27.96	1.52	0.30	47.87	0.30

N, number of samples.

*Both the genotypes contained one synonymous mutant allele TTT. The genotype F/F in SJ consisted of TTC/TTC (14.29%) and TTC/TTT (2.38%); the genotype F/S in SJ consisted of TTC/TCC (26.19%) and TTT/TCC (2.38%).

The predominant genotype in Shanghai was S/S, accounting for 47.87%, followed by F/S (27.96%), F/F (22.04%), S/C (1.52%), S/I (0.30%), and C/C (0.30%) (Table 6). The mutant homozygous genotype S/S was predominant in most populations, whereas the wild-type genotype F/F was the most frequent genotype in three populations, namely QP (55.32%), CM (65.79%), and FX (100%).

### Multiple mutations at codon 1016, 1532, and 1534

In this study, 101 individuals were identified as wild-type homozygotes. Multiple mutations were observed in all populations except FX, but no individuals with simultaneous mutations at all three codons were found.

Thirteen populations showed mutations at both codon 1016 and 1534, with three different combinations of mutations: 1016V/G+1534F/S, 1016V/G+1534S/S, and 1016G/G+1534S/S (Table 7). The most common type of combination was 1016V/G+1534F/S, with the highest frequency found in CN (17.5%, 7/40). The 1016V/G+1534S/S combination type appeared in SJ, PT, JD, HK, PD, and CN, with the highest frequency observed in PT (10.81%, 4/37). The 1016G/G+1534S/S combination type was found in only one individual from the SJ population.

**Table 7:** Simultaneous mutations at codon V1016G, I1532T, and F1534S in *Ae*. *albopictus* from Shanghai China.

Codon			Collection sites														
1016	1532	1534	CM	QP	XH	SJ	PT	YH	JS	JD	HK	MH	CX	PD	JA	YP	CN
**V/G** ^Δ^	**T/T**	**F/S** ^Δ^	3	0	0	5	5	3	4	5	1	4	4	1	5	3	7
**V/G** ^Δ^	**T/T**	**S/S** ^▲^	0	0	0	1	4	0	0	1	1	0	0	1	0	0	1
**G/G** ^▲^	**T/T**	**S/S** ^▲^	0	0	0	1	0	0	0	0	0	0	0	0	0	0	0
**V/V**	**I/T** ^Δ^	**F/S** ^Δ^	0	1	3	2	1	3	1	1	0	2	2	0	2	3	3
**V/G** ^Δ^	**I/T** ^Δ^	**F/F**	0	0	0	0	0	0	0	0	0	0	0	1	0	1	0

^Δ^, Mutant heterozygotes;

^▲^, mutant homozygotes.

No simultaneous mutations of F1534C/I or V1016G were found.

The only type of combined mutation observed at codon 1532 and 1534 was 1532I/T+1534F/S, which was found in 12 populations. The highest frequency was observed in YH (8.11%, 3/37). The only type of combined mutation at codon 1016 and 1532 was 1016V/G+1532I/T, which was observed only in PD and YP, with one individual in each population.

### Correlation analysis of introns with *kdr* genotypes in Domain III

After excluding individuals for which the exact intron length could not be determined due to sequencing peak map clutter, 638 samples with known intron lengths were analyzed in Domain III. The introns were classified into five types based on their exact lengths: intron A (83 bp), B (68 bp), C (80 bp), D (72 bp), and E (70 bp). Intron B was the most common type, representing 87.30% (557/638) of the samples, followed by intron A, representing 12.07% (77/638). Introns C, D, and E were relatively rare, accounting for only 0.16% (1/638), 0.16% (1/638), and 0.31% (2/638), respectively.

The correlation between intron types and *kdr* mutation genotypes was also explored. Intron A was predominantly found in wild-type homozygotes (1532I/I+1534F/F), representing 87.01% (67/77) of these samples (Table 8). However, intron B was primarily found in mutant homozygotes at codon 1534 (1532I/I+1534S/S), accounting for 54.94% (306/557), followed by 26.75% (149/557) in mutant heterozygotes (1532I/I+1534F/S) and 9.52% (53/557) in wild-type homozygotes (1532I/I+1534F/F). Intron C was found in only one wild-type homozygote (1532I/I+1534F/F), and intron D was also only observed in wild-type homozygotes (1532I/I+1534F/F). The two sequences containing intron E were identified in wild-type homozygotes (1532I/I+1534F/F) and mutant homozygotes (1532I/I+1534C/C), respectively.

**Table 8:** The number of individuals with introns and *kdr* mutation genotypes in Domain III.

Genotype	1532	I/I	I/I	I/I	I/I	I/I	I/T	I/T	T/T	Total
	1534	F/F	F/S	S/S	S/C	C/C	F/F	F/S	F/F	
Intron	A	67	6	3	0	0	1	0	0	77
	B	53	149	306	10	0	12	24	3	557
	C	1	0	0	0	0	0	0	0	1
	D	1	0	0	0	0	0	0	0	1
	E	1	0	0	0	1	0	0	0	2
	Total	123	155	309	10	1	13	24	3	638

The frequency of intron types in mutant individuals at codon 1534 (1532I/I) was compared. Type A introns (1.81%, 9/498) were significantly less common than Type B introns (98.00%, 488/498) in these individuals. In wild-type homozygotes (1532I/I+1534F/F, *n* = 123), 67 (54.47%) individuals possessed intron A, 53 (43.09%) possessed intron B, and the remaining 3 possessed intron C, D, and E, respectively. In mutant heterozygotes (1532I/I+1534F/S, *n* = 155), 6 (3.87%) possessed intron A, whereas 149 (96.12%) possessed intron B. In mutant homozygotes (1534S/S, *n* = 309), only 3 (0.97%) possessed intron A, whereas 306 (99.03%) possessed intron B. Statistical analysis revealed that individuals with intron B had a significantly higher mutation tendency at codon 1534 relative to those with intron A type (chi-square test, *p* < 0.0001). Regarding individuals with 1532I/T+1532T/T, the number of those with Type A introns (1/40) was significantly lower than that of those with Type B introns (39/40); however, there was no significant difference in mutation frequency between these two groups (chi-square test, *p* = 0.0916).

**Fig 3:**
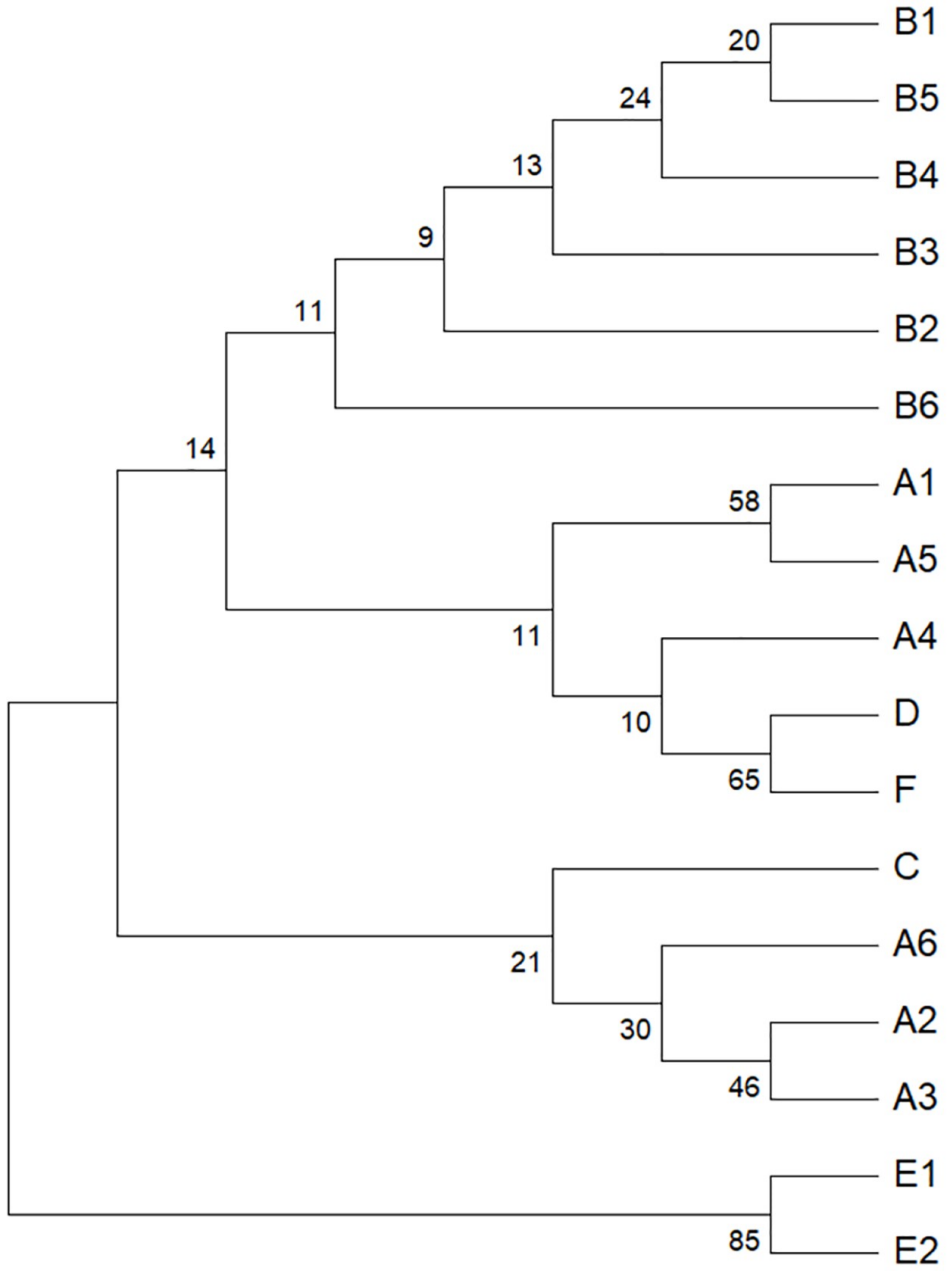
Phylogenetic tree of introns in Domain III of the *VGSC* gene in *Ae*. *albopictus* Shanghai populations.

We read 94 sequences of TA cloning products and classified them based on sequence information. Intron A had six subtypes, intron B, had six subtypes, and intron E had two subtypes. In contrast, intron C and D each had only one type of sequence. Additionally, we identified a new intron, referred to as intron F (67 bp) S1 Table). The phylogenetic tree results showed that the subtypes of intron E were distinct from the other types, and all the subtypes of intron B were grouped into a clade. In contrast, the subtypes of intron A, as well as intron C and D, clustered into another clade (Fig 3).

### Application of pyrethroids in Shanghai and its effect on *kdr* mutations

In 2021, the registered amount of pyrethroid insecticides used varied markedly among different districts in Shanghai, ranging from 26.83 g/km^2^ in Songjiang to 6006.24 g/km^2^ in Hongkou (Table 9). However, an analysis of the pyrethroid consumption and *kdr* mutation frequency in the same 14 districts did not reveal any correlation between the two variables (Pearson correlation, *p* = 0.4755). Similarly, there was no significant correlation between the frequency of allele F1534S and pyrethroid consumption (Pearson correlation, *p* = 0.2223). It should be noted that there may be some deviation between the actual amount of pyrethroid used and the registered data in some districts, as the registration of insecticide consumption was not mandatory.

**Table 9:** The area of districts in Shanghai China and the amount of pyrethroid insecticides usage.

Districts	Area (km^2^)	Amount of pyrethroid (g)	Amount of pyrethroid per unit area(g/km^2^)	*Kdr* mutation frequency (%)	Frequency of allele F1534S (%)
Fengxian	687.39	32999.52	48.01	0	0
Qingpu	670.14	223195.10	333.06	46.81	25.53
Chongming	1185.49	254808.10	214.94	82.89^a^	44.08^c^
Pudong	1210.41	133659.00	110.42	87.80^b^	68.90^d^
Yangpu	60.73	27640.00	455.13	86.96	50.00
Hongkou	23.48	141026.60	6006.24	88.10	72.62
Jiading	464.20	84737.20	182.54	95.00	71.25
Songjiang	605.64	16250.00	26.83	95.24	67.86
Changning	38.30	62000.00	1618.80	97.50	72.50
Xuhui	54.76	21250.00	388.06	100.00	88.75
Putuo	54.83	89900.00	1639.61	100.00	91.89
Minhang	370.75	25173.00	67.90	100.00	75.00
Jinshan	586.05	47506.00	81.06	100.00	82.05
Jing’an	36.88	30679.00	831.86	100.00	80.00

^a^ The value was calculated via mutation individuals of PD and YH divided by the sum of the total individuals of them;

^b^ The value was calculated via mutation individuals of CM and CX divided by the sum of the total individuals of them;

^c^ The value was calculated via the number of alleles F1534S of PD and YH divided by the sum of them;

^d^ The value was calculated via the number of alleles F1534S of CM and CX divided by the sum of them.

### Comparison of the current data with *kdr* mutation history data

The *kdr* mutations F1534S and I1532T of *Ae*. *albopictus* have been reported in previous studies [24, 36, 37], whereas V1016G has not been detected in Shanghai until now (Table 10). Although the collection sites in our study differed from those in previous studies, we observed a trend of increased F1534S frequency to some extent. In the present study, the frequencies of F1534S in Yangpu, Jiading, and Putuo in 2020 were 50.00%, 71.25%, and 91.89%, respectively, which were higher than their counterparts in previous studies. Specifically, the frequency of F1534S in Jiading district exhibited a marked increase from 46.77% in 2018 [37] to 71.25% in 2020. Furthermore, the frequency of F1534S in Putuo in 2020 was higher than that of all the five populations in 2019. The comparison between the PT and ZR populations in 2020, which were less than 1 km apart, demonstrated a significant rise in frequency from 64.58% (ZR) to 91.89% (PT) within just one year [36]. However, we found that the frequencies of the mutant allele I1532T in Yangpu, Jiading, and Putuo in 2020 were 6.52%, 1.11%, and 1.35%, respectively, which were lower than those in previous studies [24, 36, 37].

**Table 10:** The *kdr* mutation frequency of Shanghai in 2017[24], 2018[37] and 2019[36] from published information.

Year	District	Population	Frequency (%)	
			TCC/S	ACC/I
2017	Baoshan	SHBS	22.46	8.33
2017	Yangpu	SHYP	46.43	17.86
2017	Yangpu	SHGQ	36.90	17.86
2018	Jiading	SHJD	46.77	3.23
2019	Putuo	DX	64.58	12.50
2019	Putuo	CF	70.31	3.13
2019	Putuo	ZR	65.00	6.67
2019	Putuo	SD	82.81	3.13
2019	Putuo	XH	71.88	3.13

## Discussion

As a megacity with a large population and frequent flow of people and goods, Shanghai is at risk of arboviral infections such as dengue fever. However, the long-term extensive use of insecticides for mosquito-borne infectious disease prevention and control has led to mosquito resistance, posing a risk of failure in eradication efforts. To address this issue, we conducted a study to detect *kdr* mutations in *Ae*. *albopictus* populations in Shanghai in 2020. Our results revealed an unexpectedly high frequency of *kdr* mutations. Furthermore, V1016G and F1534C were detected in Shanghai for the first time, and a novel mutation, F1534I, was discovered.

Bioassays were previously conducted to test the susceptibility of *Ae*. *albopictus* in several districts in Shanghai. The results indicated that most populations exhibited different degrees of resistance to pyrethroids. Specifically, in 2017–2018 [38], populations from Pudong, Huangpu, Jing’an, Songjiang, Hongkou, Yangpu, and Minhang were moderately resistant to deltamethrin, whereas those in Chongming, Baoshan, Jinshan, and Xuhui were highly resistant to deltamethrin. Additionally, populations from Qingpu showed low resistance to beta-cypermethrin, whereas those from Yangpu, Hongkou, Songjiang, Minhang, Jinshan, Putuo, Huangpu, and Pudong were moderately resistant, and those from Chongming, Jing’an, Xuhui, Fengxian, and Baoshan were highly resistant. Finally, populations from Yangpu and Jinshan were moderately and highly resistant to permethrin, respectively. In a separate study, larvae in Xuhui exhibited increased resistance to beta-cypermethrin, showing moderate resistance in 2019 but high resistance in 2021 [39]. Regarding adult mosquitoes, *Ae*. *albopictus* in Yangpu and Baoshan were found to be resistant to permethrin, deltamethrin, beta-cypermethrin, and lambda-cyhalothrin [30, 40]. Additionally, adults in Jiading were resistant to beta-cypermethrin and deltamethrin, whereas those in Qingpu were resistant to deltamethrin and permethrin [41, 42].

Based on previous studies, *kdr* mutations such as F1534S and I1532T have been found in several districts in Shanghai, including Baoshan, Yangpu, Putuo, and Jiading [24, 36, 37]. In the present study, we found an increased frequency of the F1534S mutation relative to that in historical data, suggesting rapid evolution of *kdr* mutations in *Ae*. *albopictus* populations in Shanghai. These mutations raise concerns about the effectiveness of mosquito-borne disease prevention and control methods, particularly for diseases such as dengue fever.

Multiple *kdr* mutations, including F1534S, F1534C, and I1532T, have been linked to pyrethroid resistance in *Ae*. *albopictus*. Bioassay studies have shown that the F1534S mutation is positively correlated with deltamethrin and permethrin resistance [30]. Additionally, introducing the I1532T and F1534S/L mutations separately or together into the pyrethroid-sensitive sodium channel AaNav1-1 via site-directed mutagenesis has been found to substantially reduce channel sensitivity to Type I pyrethroids (permethrin and bifenthrin) but not to Type II pyrethroids (deltamethrin and cypermethrin) [43]. Furthermore, F1534S plays a more important role in pyrethroid resistance than F1534L or F1534C, and the I1532T mutation has little effect on deltamethrin and sodium channels [43]. Although some studies have found a positive correlation between both the I1532T and F1534S mutations and deltamethrin resistance [25], others have found a negative correlation between the I1532T mutation and this phenotype [30]. Bioassays conducted in combination with genotyping have shown that the I1532T allele frequency in adults is related to pyrethroid resistance, but no correlation was found between I1532T and deltamethrin resistance [44]. Overall, it appears that I1532T is a *kdr* mutation that contributes to faint resistance to permethrin in *Ae*. *albopictus*.

Several electrophysiological studies have suggested that the F1534C mutation confers resistance to Type I pyrethroids but not to Type II pyrethroids [45, 46]. However, bioassay studies have shown that F1534C exhibits resistance to both types of pyrethroids [47, 48]. Additionally, the V1016G allele provides resistance to both Type I and Type II pyrethroids, as shown by electrophysiological studies and bioassays [45, 47, 48]. Identification of the V1016G mutation in *Ae*. *albopictus* populations in Shanghai, along with an increase in *kdr* mutation types, suggests a significant rise in resistance to pyrethroids. Furthermore, the discovery of the F1534I mutation in only two samples in Shanghai requires further investigation to determine its contribution to pyrethroid resistance in *Ae*. *albopictus*.

The extensive use of insecticides is believed to be a key factor contributing to the rapid evolution of *kdr* mutations. However, our analysis did not find any correlation between mutation frequencies and the amount of insecticide consumed, likely because there is no mandatory registration of pyrethroid usage. This highlights the inadequacy of current insecticide management and the urgent need for its improvement.

Among all the samples, the FX population is particularly noteworthy owing to its absence of *kdr* mutations, with a mutation frequency of 0%. Given this surprising result, we conducted further sampling on September 30, 2022 at the same collection sites, and conducted a detailed survey of the surrounding environment. We extracted and sequenced DNA from 30 adult female mosquitoes and found only one I1532T mutation heterozygote, confirming the absence of *kdr* mutations in the FX population. We also found that the breeding sites were primarily located along a karting track in the Shanghai Bay National Forest Park where no insecticide had been used, potentially resulting in few *kdr* mutations in the FX population. The surrounding forest may also insulate *Ae*. *albopictus* populations from the effects of nearby insecticide use, contributing to their relative geographic isolation. The presence of this population without *kdr* mutations underscores the need to restrict insecticide use to slow the development of resistance.

The mechanism of *kdr* mutation has been a topic of interest in recent years. Through the data analysis in the present study, we found that introns might play a role in influencing *kdr* mutation frequency. However, research on introns in the *VGSC* gene of *Ae*. *albopictus* is scarce. Based on research related to *Ae*. *aegypti*, the intron separating codon 1011 (exon 20) and 1016 (exon 21) in the *VGSC* genomic region of *Ae*. *aegypti* has been identified as a potential factor affecting *kdr* mutation frequency. This intron has been classified into two groups, A (250 bp) and B (234 bp) [49], and I1011M and V1016I have been found to coexist with group A in *Ae*. *aegypti* in Brazil [49], S989P, V1016G, and D1763Y have been found to coexist with group A in *Ae*. *aegypti* in Taiwan [50], and F1534C has been found in group B in *Ae*. *aegypti* in Africa and Taiwan [50, 51]. Therefore, there could be a relationship between *kdr* mutations and introns in *VGSC* that warrants further investigation. However, in a previous study, the intron at the same codon in *Ae*. *albopictus* was found to be much shorter than that in *Ae*. *aegypti*, with four different introns consisting of 81, 81, 84, and 90 bp, respectively [52]. In addition, five different introns were found in Domain III, of which two introns consisted of 68 and 70 bp, respectively, and the other three introns each consisted of 83 bp [52]. In the present study, introns A, B, and E were consistent with those in this previous study [52], although two other introns, referred to as C and D, were also found. The types of introns appear to play an important role in the regulation of gene mutation, but how this effect occurs remains unclear. Therefore, it is important to investigate the possible mechanisms underlying the effects of different intron types on *kdr* mutations.

Finally, it is worth noting that there are some limitations to our study. In addition to *kdr* mutations, metabolic resistance, reduced penetration, and behavioral resistance all contribute to resistance mechanisms [14]. This study only discussed the *kdr* resistance of *Ae*. *albopictus* in Shanghai, ignoring the effect of other factors, particularly metabolic resistance. Moreover, the introns in this study were classified according to their length, whereas the distinctions among the sequences of the introns were not explored and therefore require further study.

## Conclusions

The frequency of *kdr* mutations in *Ae*. *albopictus* in Shanghai, China was found to be alarmingly high, reaching 84.65%. Notably, the mutant alleles V1016G and F1534C were detected for the first time in Shanghai, and a novel mutation, F1534I, was also identified. Our study found that intron B in Domain III was significantly associated with the mutation frequency at codon 1534. This rapid evolution of *kdr* mutations is concerning and highlights the increasing risk of mosquito-borne disease outbreaks. Therefore, it is crucial to continuously monitor resistance changes and implement strict regulations on insecticide use to slow the development of resistance.

## Supporting information

S1 Table Sequence comparison of introns of *VGSC* gene in Domain III. (XLSX)
